# Comparison of Nutritional Profiles and Antioxidant Capacity in the Muscle of *Procambarus clarkii* Farmed Across Various Rice Paddy Regions in Eastern China

**DOI:** 10.3390/antiox15070887

**Published:** 2026-07-17

**Authors:** Linjun Zhou, Chonghang Ding, Rui Jia, Yiran Hou, Chengfeng Zhang, Liqiang Zhang, Bing Li, Jian Zhu

**Affiliations:** 1 Key Laboratory of Integrated Rice-Fish Farming Ecology, Ministry of Agriculture and Rural Affairs, Freshwater Fisheries Research Center, Chinese Academy of Fishery Sciences, Wuxi 214081, China; zhoulinjun@ffrc.cn (L.Z.);; 2 Shandong Fishery Development and Resource Conservation Center, Jinan 250014, China; 3 Wuxi Fisheries College, Nanjing Agricultural University, Wuxi 214081, China

**Keywords:** *Procambarus clarkii*, integrated rice–crayfish co-culture, antioxidant capacity, amino acids, fatty acids, trace elements, metabolic profiles

## Abstract

The aim of the study was to assess the regional differences in *Procambarus clarkii* via analyzing amino acid and fatty acid profiles, minerals, antioxidant capacity, and metabolic profiles from ten major producing areas in China. The results showed that *P. clarkii* cultured in rice fields was a high-quality source of amino acids and fatty acids. The *P. clarkii* collected from Feixi and Suqian city exhibited higher amino acids and polyunsaturated fatty acids, whereas those from Honghu and Jianli city displayed lower unsaturated fatty acids. Mineral profiles showed clear regional heterogeneity, with K and Na as the predominant elements; Feixi city was characterized by relatively high K and Mg levels, while Nanxian and Honghu city showed higher Zn and Fe accumulation. Moreover, antioxidant-related indicators showed region-specific and mixed patterns, indicating that antioxidant status should be interpreted based on multiple biomarkers rather than any single indicator. Metabolomic profiling also revealed clear geographic clustering, with regional differences mainly associated with amino acid and lipid metabolism. Overall, the nutritional composition and antioxidant capacity of *P. clarkii* muscle exhibited clear regional variability. Our findings provide a theoretical basis for quality evaluation in *P. clarkii* aquaculture and offer practical guidance for refining the development models and strategic direction of integrated rice–crayfish farming across different regions.

## 1. Introduction

*Procambarus clarkii* is one of the most economically important freshwater crustaceans in China. Since its introduction in the 1930s, this species has rapidly expanded in aquaculture because of its strong environmental adaptability, omnivorous feeding habit, high reproductive capacity, and suitability for shallow paddy-field habitats [1,2]. In the rice–crayfish co-culture system, both rice and crayfish are cultivated concurrently in the same paddy field, sharing the same ecological resources, including water and soil. Numerous research indicate that this model not only enhances soil fertility but also leads to a reduction in the use of nitrogen and phosphorus fertilizers [3]. Furthermore, the presence of crayfish in these paddies allows for the utilization of natural food sources available in the rice fields, thereby decreasing the dependence on artificial feed inputs [4]. Consequently, the rice–crayfish co-cultivation approach has emerged as the predominant model for *P. clarkii* farming in China [5].

Muscle tissue represents the primary edible component of aquatic animals and determines their nutrient composition. Specifically, amino acids are fundamental components of muscle protein and contribute to both human dietary value and taste characteristics, whereas fatty acids, particularly polyunsaturated fatty acids, are closely related to nutritional benefits and consumer acceptance [6]. Minerals such as K, Na, Ca, Mg, Zn, Fe, Cu, and Mn are also important indicators of the nutritional value and physiological status of aquatic products [7]. Hence, enhancing muscle quality is integral to meeting the standards of food quality. However, the muscle quality of aquatic animals varies significantly due to farming methods and environmental conditions. Previous research has revealed noticeable difference in the composition of fatty acids and free amino acids between wild and farmed *Dicentrarchus labrax* [8]. Further comparison across various farming environments revealed that *Eriocheir sinensis* from Yangcheng Lake demonstrated a more desirable flavor than those from other regions [9]. Compared with the crayfish in other culture systems, the nutritional value and flavor of muscle were found to be better in an integrated rice–crayfish farming system [10,11]. For instance, a study found that pond-cultured individuals had higher meat yield, protein and lipid contents, essential amino acid levels, and n-3/n-6 PUFA ratios, whereas rice-field-cultured crayfish were characterized by higher trace mineral contents and also exhibited favorable nutritional quality [12]. Similarly, Wang et al. compared *P. clarkii* from pond intensive culture, rice–crayfish co-culture, cement pond culture, and wild habitats, and reported that the rice–crayfish co-culture improved the nutritional and taste-related quality of muscle amino acids and fatty acids, while cement pond culture was associated with higher crude protein content and nonspecific immunity [13]. These findings suggest that culture environment can strongly influence crayfish muscle quality, but the reported effects vary among culture modes and quality indicators. However, reports on the nutritional value of crayfish muscle in rice–fish integrated farming systems across different regions remain limited.

Antioxidant capacity is another important quality-related indicator because aquatic animals are frequently exposed to environmental fluctuations, including changes in temperature, dissolved oxygen, feeding conditions, and stocking density [14]. Organisms can produce an excess of reactive oxygen species (ROS) when experiencing various environmental stresses, such as fluctuations in water temperature, dissolved oxygen levels, feeding routines, or farming density [15]. Typically, these ROS can be neutralized by the organism’s antioxidant defense system. Proper external stimuli may activate the immune and antioxidant responses of organisms, thereby enhancing their antioxidative capacity, improving stress resilience, and bolstering immunity [16]. However, exceeding the organism’s tolerance threshold can lead to oxidative damage to cells, tissues, and even the organs, which may trigger outbreaks of disease and potentially result in mortality. For example, acute hypoxia/ reoxygenation stress could cause the disturbance of antioxidant enzymes, lipid peroxidation, and a decrease in immune functions in *P. clarkii* [17]; high farming density resulted in decreased antioxidant capacity and abnormal lipid metabolites in *Pelteobagrus fulvidraco* [18]. Therefore, comparing antioxidant indicators among regions is useful for evaluating the physiological quality and stress status of crayfish cultured under different production conditions.

Although rice–crayfish farming has expanded rapidly in China, the quality characteristics of *P. clarkii* from different major producing regions remain insufficiently characterized. In particular, it remains unclear whether regional differences in muscle nutritional composition and antioxidant capacity are accompanied by changes in metabolic profiles, and how metabolomic variation relates to amino acid and lipid metabolism. Addressing this research gap, we sourced *P. clarkii* specimens from ten leading production areas throughout China. We then conducted comprehensive analyses to determine the profiles of amino acids, fatty acids, minerals, antioxidant capacities, and metabolites within the muscle tissue. This study aims to provide a comprehensive basis for evaluating the nutritional quality of *P. clarkii* muscle and offers practical guidance for regional quality assessment and the selection of areas better suited for rice–crayfish farming.

## 2. Materials and Methods

### 2.1. Sample Collection and Pretreatment

**Figure 1:**
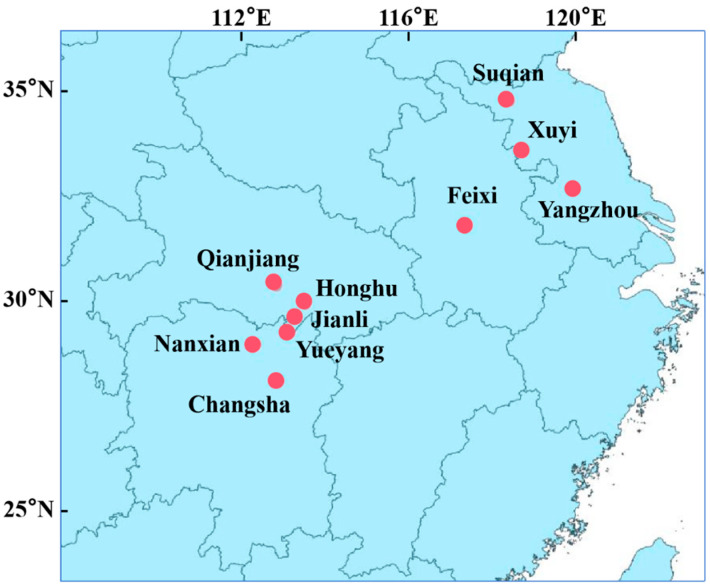
Geographic map of the sampling area of *P. clarkii* in China.

In the present study, specimens of *P. clarkii* were collected from ten integrated rice–crayfish farming systems located across four major producing provinces in China, including Feixi County (FX) in Hefei, Anhui Province; Suqian (SQ), Yangzhou (YZ), and Xuyi (XY) in Huaian City, Jiangsu Province; Qianjiang (QJ), Jianli (JL), and Honghu (HH) in Jingzhou City, Hubei Province; and Yueyang (YY), Changsha (CS), and Nanxian (NX) in Yiyang City, Hunan Province (Figure 1). All samples were collected during the same production season from 20 June to 10 July in same year, to minimize the potential influence of seasonal variation. During the experiment, commercial feed (Hailong Co., Ltd., Maoming,China) was fed twice daily. The main nutrient composition of the diet included 30.0% crude protein, 3.0% crude fat, 8.0% crude fiber, and 18.0% crude ash. Feeding conditions were consistent in all regions. A total of 200 *P. clarkii* specimens were collected from each region, with a sex ratio of approximately 1:1. For each region, the 200 individuals were randomly divided into 10 independent pool; each pooled replicate consisted of muscle tissues from 20 individuals, including 10 males and 10 females, to minimize the potential influence of sex ratio on regional comparisons. The average weight of the collected *P. clarkii* was 31.90 ± 6.71 g. All collected samples were freeze-dried, ground into powder, and immediately stored at −80 °C pending analysis.

### 2.2. Amino Acid and Fatty Acid Analyses

The composition of amino acids in muscle tissue was quantified using an acid hydrolysis method in accordance with the Chinese standard GB5009.124-2016. The freeze-dried muscle samples were hydrolyzed at 110 °C for 22 h with 10 mL of 6 mol/L hydrochloric acid. After filtration, 0.25 mL of the hydrolysate was dried under pressure, and then re-dissolved in a 0.02 mol/L hydrochloric acid solution and filtered again. Finally, the samples were analyzed using an LA8080 Automatic Amino Acid Analyzer (Hitachi, Japan) with wavelengths of 570 nm and 440 nm, and a reaction temperature of 135 °C.

The fatty acid composition of the samples was determined in accordance with the GB5009.168-2016 standard in China. The samples were hydrolyzed, saponified, and esterified before being analyzed using an Agilent 7890 A gas chromatograph equipped with a TG-FAME chromatographic column (50 m × 0.25 mm × 0.20 μm). The inlet temperature was set at 270 °C, and the detector FID temperature was set at 280 °C. N2 was used as the carrier gas at a flow rate of 0.63 mL/min, and the shunt ratio was set at 100:1. All determinations were performed in quadruplicate to ensure accuracy.

### 2.3. Mineral Analyses

The freeze-dried muscle tissue samples of *P. clarkii* were added with HNO_3,_ and microwave digestion was performed after the reaction was complete by using TOPEX microwave digestion apparatus (Shanghai Yiyao, China). The digests were cooled and diluted to 25 mL with ultrapure water. Blank samples used the same method. The contents of minerals in digestives were determined by inductively coupled plasma mass spectrometry (ICP-MS; iCAPRQ, USA). The content was expressed as g/kg dry weight for K, Na, Mg, and Ca; the content was expressed as mg/kg dry weight for Zn, Fe, Cu, and Mn; the content was expressed as μg/kg dry weight for Y.

### 2.4. Antioxidant Capacity Analyses

A total of 0.05 g freeze-dried muscle tissue sample was added with 0.95 mL of normal saline (0.86%) to make 5% muscle homogenate. A range of parameters related to antioxidant capacity were measured using commercial kits, including total antioxidant capacity (T-AOC), superoxide dismutase (SOD), glutathione (GSH), malondialdehyde (MDA), catalase (CAT), and glutathione peroxidase (GSH-Px). SOD and GSH kits were purchased from Nanjing Jiancheng Bioengineering Institute (Nanjing, China); MDA and CAT were ordered from Beyotime Biotechnology Co., Ltd. (Shanghai, China); T-AOC and GSH-Px were provided from Grace Biotechnology Co., Ltd. (Suzhou, China). All parameters were conducted according to the kit instructions.

### 2.5. Untargeted Metabolomic Profiling

Tissue samples (80 mg) were taken, homogenized, and then suspended in 80% methanol under ice-water bath conditions. Following centrifugation, the resulting supernatant was diluted to achieve a final concentration of 53% methanol. The obtained supernatant was directly analyzed using UHPLC (1290 Infinity LC, Agilent Technologies), coupled to a quadrupole time-of-flight (AB Sciex TripleTOF 6600) in Gene Denovo Biotechnology Co., Ltd. (Guangzhou, China) (see Supplementary Materials for details). The generated raw data were processed using Compound Discoverer 3.1 (CD3.1, Thermo Fisher Scientific, Waltham, M, USA) for metabolite quantitation. Metabolite annotation was carried out by querying the KEGG, HMDB, and LIPIDMaps databases. Multivariate statistical analyses, including principal component analysis (PCA) and partial least squares-discriminant analysis (PLS-DA), were performed on the metabolomic dataset using the metaX package(version 1.4.2). Univariate analysis was performed to calculate statistical significance, and the resulting *p*-values were corrected for multiple comparisons using the Benjamini–Hochberg false discovery rate (FDR) method. The corrected values were reported as q-values or FDR-adjusted *p*-values. Metabolites with variable importance in projection (VIP) > 1 and q-value (FDR-adjusted *p*-value) < 0.05 were considered significantly differential.

### 2.6. Statistical Analysis

The original data were preliminarily sorted out by Excel software and analyzed by SPSS 27.0 software (IBM Corp., Armonk, NY, USA). One-way analysis of variance (ANOVA) with post hoc LSD was used to compare significant differences between different groups (SPSS 27.0). The values are expressed as mean ± SE (standard error). Statistical significance was set at *p* < 0.05. The results were plotted using GraphPad Prism 8.0 and Adobe Illustrator 2024 software.

## 3. Results

### 3.1. Amino Acid Composition

Seventeen amino acids were identified in the muscle tissue of *P. clarkii* across various locations (Table 1), including 7 essential amino acids (EAA), 2 half-essential amino acids (HEAA), and 8 non-essential amino acids (NEAA). Glutamate (Glu), arginine (Arg), and aspartate (Asp) predominate in the muscle’s amino acid profile. Within the EAAs, lysine (Lys) was the most abundant. The EAA percentage ranged from 36.7% in the FX area to 38.1% in HH. FX specimens exhibited the highest TAA and EAA levels among the surveyed areas, exceeding the levels found in other groups (*p* < 0.05). Samples from SQ, CS, and NX regions also exhibited higher amino acid concentrations. In contrast, HH specimens had the lowest TAA and EAA concentrations, a trend similarly observed in JL and YY regions. Apart from cysteine (Cys), *P. clarkii* from FX had the highest levels of all detected amino acids, with histidine (His), serine (Ser), tyrosine (Tyr), glycine (Gly), and aspartate (Asp) being notably higher compared to most groups (*p* < 0.05). In contrast, HH samples exhibited the lowest concentrations of amino acids except for Arg, with phenylalanine (Phe), isoleucine (IIe), valine (Val), Cys, and proline (Pro) significantly lower than in other areas (*p* < 0.05). These data indicated that marked geographic variation in amino acid profiles was evident across the different locations.

### 3.2. Fatty Acid Composition

The fatty acid composition of *P. clarkii* muscle from various regions is presented in Table 2. PUFAs constituted the highest proportion, exceeding one-third of the total fatty acid content. Among 21 fatty acids, C18:1n9c was the most abundant fatty acid, exceeding 20%, followed by C16:0, C18:0, C20:5n3, and C18:2n6. Fatty acid contents were highest in specimens from FX, moderately high in NX and SQ, and notably lowest in JL and HH, with a significant difference compared to other regions (*p* < 0.05).

Among the nine SFAs, C16:0 and C18:0 were the predominant SFAs. SFA contents were highest in NX, followed by XY, YY, and FX in descending order. In contrast, JL exhibited the lowest levels of SFA, which were significantly lower than those recorded in the aforementioned locations (*p* < 0.05). Except C14:0, the content of eight SFAs in JL was significantly lower than that in other areas (*p* < 0.05). The monounsaturated fatty acid (MUFA) levels in YZ, JL, and HH were significantly lower compared to other regions, with JL exhibiting the lowest MUFA content (*p* < 0.05). Specifically, the C16:1 level in FX, SQ, and XY, as well as the C20:1 level in CS and NX, were significantly higher. Conversely, the C16:1 levels in HH and NX, the C18:1n9c levels in YZ, JL, and HH, and the C20:1 levels in SQ, YZ, and JL were significantly reduced when compared to other areas (*p* < 0.05).

Among the eight PUFAs, C18:2n6 and C20:5n3 were the predominant forms. The PUFA content was found to be the highest in FX, whereas JL and HH exhibited significantly lower concentrations (*p* < 0.05). Additionally, except for C20:3n6, the concentrations of the seven remaining PUFAs in JL were significantly lower compared to those measured in other regions (*p* < 0.05). The n-3/n-6 ratio varied from 0.92 in NX to 1.42 in CS, surpassing 1 in all sampled regions except for NX. Within the ten distinct regions, the content of n-3 PUFAs was found to be higher in FX and SQ while it was lower in JL and HH, a variation that was statistically significant (*p* < 0.05). Similarly, n-6 PUFA levels were elevated in NX, yet significantly diminished in JL, HH, and CS when compared to other regions (*p* < 0.05). The arachidonic acid (AA) content was higher in NX and YZ, but lower in XY and JL. The combined content of docosahexaenoic acid (DHA) and eicosapentaenoic acid (EPA) was elevated in SQ, whereas it was reduced in YZ, HH, and JL, with these differences being statistically significant (*p* < 0.05). Furthermore, the content of EPA was found to be greater than that of DHA.

### 3.3. Mineral Composition

**Figure 2:**
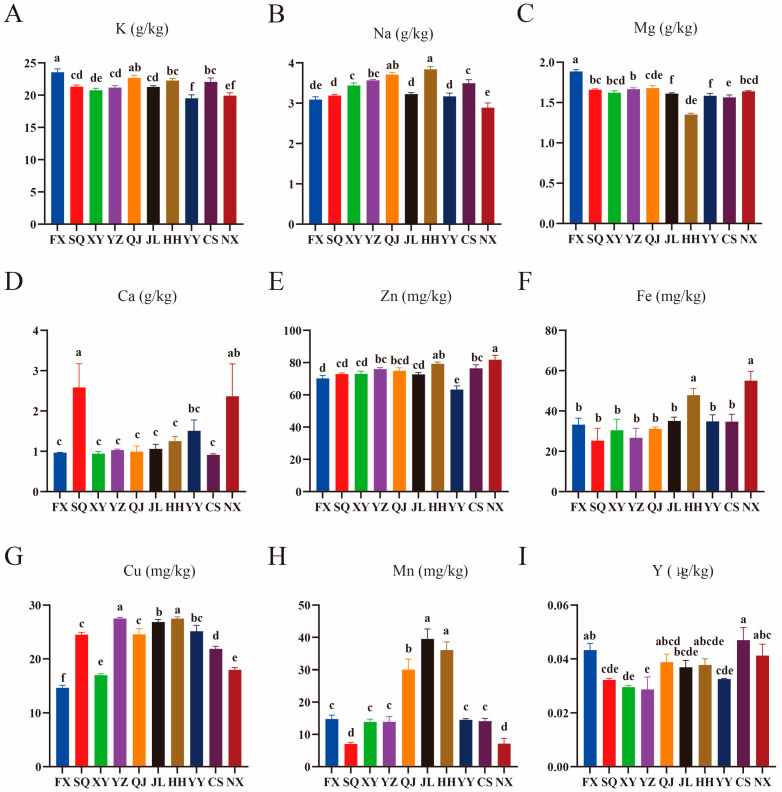
The mineral concentrations in the muscle of *P. clarkii* from different regions (dry matter). (**A**–**D**) macroelement; (**E**–**H**) trace element; (**I**): rare earth element. Values are presented as means ± SE (*n* = 10). Different letters in the same index indicated significant difference (*p* < 0.05). FX, Feixi County; SQ, Suqian city; YZ, Yangzhou city; XY, Xuyi County; QJ, Qianjiang city; JL, Jianli County; HH, Honghu city; YY, Yueyang city; CS, Changsha city; NX, Nanxian County.

A total of nine minerals were detected in the muscle tissue of *P. clarkii* collected from different regions, including four macroelements (Ca, Mg, Na and K), four trace elements (Fe, Cu, Mn and Zn), and the rare earth element Y (Figure 2). Overall, the mean mineral concentrations followed the order K > Na > Mg > Ca > Zn > Fe > Cu > Mn > Y, indicating that K and Na were the predominant mineral elements in *P. clarkii* muscle. The contents of K in FX and QJ were significantly higher than that in other areas, whereas the contents in NX and YY were notably lower (*p* < 0.05). The contents of Na were significantly higher in HH, QJ, and XY (*p* < 0.05). The content of Mg in FX was significantly higher, whereas HH and CS exhibited significantly lower levels (*p* < 0.05). The contents of Ca were significantly higher in SQ and NX (*p* < 0.05). The contents of Zn in NX and HH were significantly higher than that in other areas, while in YY, was significantly lower (*p* < 0.05). The contents of Fe were the highest in NX and HH (*p* < 0.05), with no significant differences observed among the other areas. The contents of Cu in XY, HH, and JL were significantly higher, while in FX, YZ, and NX, were significantly lower (*p* < 0.05). The contents of Mn in JL, HH, and QJ regions were significantly higher, while in NX and SQ, were significantly lower (*p* < 0.05). Y was more abundant in CS and FX, but less in XY and YZ. Collectively, these results demonstrate pronounced regional heterogeneity in the mineral composition of *P. clarkii* muscle, suggesting that culture practices may influence mineral accumulation patterns. These findings also indicate that mineral accumulation patterns vary among production regions and warrant further investigation together with water, sediment, soil, and management-related factors.

### 3.4. Antioxidant Capacity

**Figure 3:**
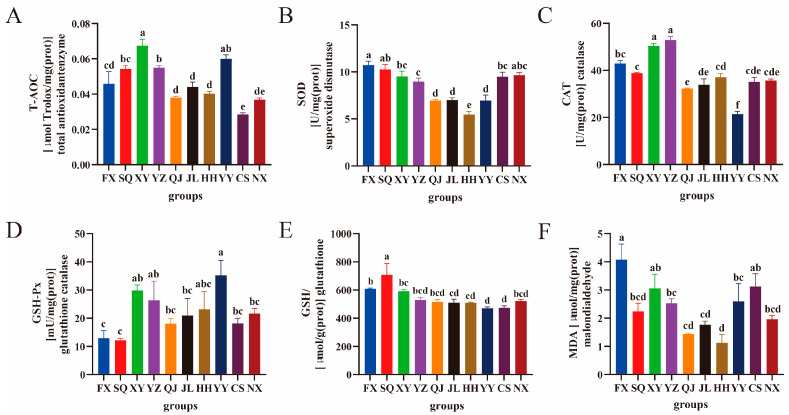
Antioxidant capacity of *P. clarkii* from different areas. Values are presented as means ± SE (*n* = 10). Different letters in the same index indicated significant difference (*p* < 0.05). (**A**) total antioxidant capacity (T-AOC), (**B**) superoxide dismutase (SOD), (**C**) catalase (CAT), (**D**) glutathione peroxidase (GSH-Px), (**E**) glutathione (GSH), (**F**) malondialdehyde (MDA). FX, Feixi County; SQ, Suqian city; YZ, Yangzhou city; XY, Xuyi County; QJ, Qianjiang city; JL, Jianli County; HH, Honghu city; YY, Yueyang city; CS, Changsha city; NX, Nanxian County.

As shown in Figure 3, the level of T-AOC in XY and YY was significantly higher than that of other regions, while CS exhibited a lower level (*p* < 0.05). SOD activities in FX and SQ, alongside CAT activities in YZ and XY, were significantly higher compared to other regions. Conversely, both SOD and CAT activities in QJ and YY were significantly lower than those in the other areas (*p* < 0.05). GSH-Px activity was significantly higher in YY (*p* < 0.05), and also higher in XY and YZ, while being lower in SQ and FX. The GSH content exhibited minimal variation across all groups; however, the GSH content in SQ was significantly higher than in the others (*p* < 0.05). MDA content was notably highest in FX (*p* < 0.05), with increased levels observed in CS and XY as well. Conversely, HH displayed the lowest MDA content (*p* < 0.05), with QJ and JL also presenting reduced levels. Overall, XY, YY, SQ, YZ, and FX showed relatively high values for specific antioxidant-related biomarkers, but these patterns were not consistent across all indicators. Our results indicated that antioxidant-related indicators displayed region-specific and mixed patterns among the ten regions.

### 3.5. Metabolic Profile

**Figure 4:**
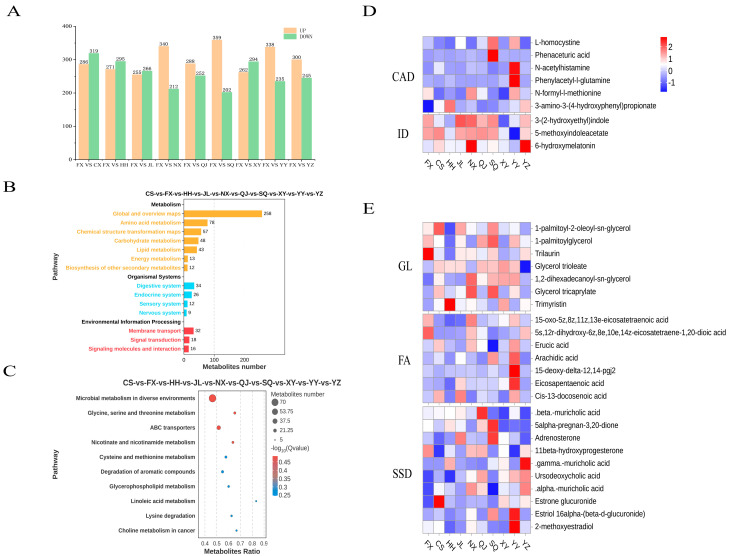
Metabolite profiles of muscles of *P. clarkii* from ten areas. (**A**) Intergroup variation in differential metabolites, (**B**) KEGG pathway enrichment of differential metabolites among areas, (**C**) bubble plot of enriched metabolic pathways across different regions, (**D**) differential metabolites related to amino acid (CAD, carboxylic acids and derivatives; ID, indoles and derivatives), (**E**) differential metabolites related to lipids and lipid-like molecules (GL, glycerolipids; FA, fatty acyls; SSD, steroids and steroid derivatives).

To complement the biochemical assessment of muscle quality, untargeted metabolomic profiling was further used to characterize the metabolic features underlying regional differences in nutritional composition and antioxidant-related indicators. Quality control analysis (QC) and OPLS-DA results revealed the clear clustering pattern in the PCA score plot, with samples from identical geographic origins grouping together and those from disparate regions exhibiting pronounced segregation (Figure S1). This pattern supported the technical reliability of the sequencing data and underscores significant inter-regional variations in the muscle metabolome. Based on the nutritional assessment conducted in this study, the FX population showed a comparatively superior muscle nutritional profile, as reflected by higher total amino acid, essential amino acid, total fatty acid, and polyunsaturated fatty acid contents. In addition, FX also exhibited relatively high SOD activity among the antioxidant indicators. Therefore, FX was selected as a representative high-nutritional-quality group and used as the reference group for pairwise metabolomic comparisons. It should be noted that FX was not considered an experimental control group, but rather a reference population used to facilitate the identification of region-specific metabolomic differences among the remaining groups. A total of 1848 metabolites were annotated across all samples, with 367 organic acids and derivatives representing the largest metabolite class, followed by 338 organoheterocyclic compounds, and 336 lipids and lipid-like molecules. Pairwise comparisons with FX revealed substantial region-specific metabolomic variation, comprising 255–359 increased and 202–319 decreased metabolites across the remaining regions (Figure 4A). Both intergroup (Figure 4B,C) and pairwise comparisons (Figures S2 and S3) showed that regional differences were mainly reflected in amino acid, lipid, and energy metabolism. CS, HH, JL, and NX were characterized by shifts in amino acid metabolism, whereas CS, JL, QJ, and XY showed more pronounced changes in lipid metabolism, particularly glycerolipid and glycerophospholipid metabolism. SQ, YY, and YZ were mainly enriched in energy-related pathways, including the pentose phosphate pathway and oxidative phosphorylation.

In amino acid metabolism, 6 carboxylic acids and their derivatives and 3 indoles and their derivatives showed pronounced region-specific variation (Figure 4D). The SQ group was characterized by relatively higher levels of L-homocystine and phenaceturic acid (*p* < 0.05), whereas the YY group showed enrichment of N-acetylhistamine and phenylacetyl-L-glutamine (*p* < 0.05). In contrast, the HH group was mainly distinguished by an increased abundance of 3-amino-3-(4-hydroxyphenyl) propionate (*p* < 0.05). For indoles and their derivatives, NX and YZ showed relatively higher levels of 6-hydroxymelatonin (*p* < 0.05). By comparison, HH and YY exhibited generally lower levels of indole-related metabolites, while XY was characterized by a marked decrease in 3-(2-hydroxyethyl) indole (*p* < 0.05). For the lipids and lipid-like molecules, significant differences were observed in 7 glycerolipids, 7 fatty acyls, and 10 steroids and steroid derivatives (Figure 4E). The HH group was characterized by the relative enrichment of trimyristin, whereas the NX group showed increased levels of 1,2-dihexadecanoyl-sn-glycerol and glycerol tricaprylate. In contrast, CS, JL, and SQ exhibited higher abundances of selected monoacylglycerol and diacylglycerol species, indicating region-specific variation in glycerolipid metabolism. In addition, YY was distinguished by higher abundances of arachidic acid, 15-deoxy-delta-12,14-PGJ2, and eicosapentaenoic acid, while NX showed increased levels of several fatty acids and their derivatives. Several steroids and steroid derivatives were significantly enriched in QJ, YY, YZ, SQ, and CS. Moreover, multiple phosphatidylcholine (PC) and phosphatidylethanolamine (PE) were relatively enriched in YY, HH, and JL, whereas several glycerophospholipids showed lower abundances in CS, QJ, and SQ (Figure S4). Overall, the metabolomic results were broadly consistent with the regional differences observed in amino acid and fatty acid profiles. The variation in amino acid-related metabolites and enriched amino acid metabolic pathways supported the differences in total and essential amino acid contents among regions. Similarly, the changes in glycerolipids, fatty acyls, phosphatidylcholines, and phosphatidylethanolamines were in line with the regional variation in fatty acid composition, particularly the differences in PUFA, n-3 PUFA, and DHA/EPA levels. These findings indicate that the metabolomic profiles provided complementary biochemical information for interpreting regional differences in *P. clarkii* muscle quality.

## 4. Discussion

### 4.1. Regional Variations in Muscular Amino Acid Profiles

The type and content of amino acids are crucial indicators for assessing the protein quality of aquatic products [19]. A high-quality protein source should not only have a diverse range of amino acids but also maintain an appropriate proportion. The variation in protein and amino acid content within the same species can be influenced by factors such as diet, age, environment, or the season of capture [20]. According to the reference protein standards formulated by the Food and Agriculture Organization of the United Nations (FAO) and the World Health Organization (WHO) [21], an optimal nutritional profile for food is characterized by EAA/TAA of about 40%, and the ratio of EAA/NEAA is more than 60%. The average EAA/TAA in different areas was from 36.70% (FX) to 38.10% (HH), closely aligning with the 40% benchmark. These ratios were higher than those found in cement pond culture (35.35%) and crab–crayfish co-culture modes (34.90%) [13]. This data indicates that meat from the crayfish in rice–crayfish co-culture is a high-quality protein source capable of satisfying human nutritional requirements.

The results also showed regional variations in the amino acid composition of *P. clarkii* muscle, suggesting that the rearing environment influences the amino acid profile, which in turn affects muscle quality. Previous studies have shown that the muscle elasticity, protein, and EAA contents of *P. clarkii* in Suqian were higher than those in Nanjing and Suzhou [22]. Similarly, *P. clarkii* from Wuhan exhibit greater muscle protein, EAA, and antioxidant capacity compared to those from Shaoguan [23]. Furthermore, *P. clarkii* from Hubei and Anhui exhibit greater muscle EAA and mineral contents compared to those from Jiangsu [24]. In addition, other factors, such as different farming models, can also cause alterations in the amino acid profile. The *P. clarkii* muscle crude protein content in integrated rice–crayfish culture model was different from that cultured in ponds or the wild [12]. In this study, *P. clarkii* from the FX, CS, and SQ regions exhibited higher amino acid content, suggesting an enhanced nutritional value. Variations in the amino acid profiles across different regions may be attributed to factors such as aquaculture practices, water quality, microbial communities in the water bodies, and temperature variations. The detailed mechanisms underlying these differences warrant further investigation.

### 4.2. The Effects of Farming Condition on Fatty Acid Composition

Fatty acids play a crucial role in influencing muscle quality, as they participate in an array of physiological processes. SFA is an important energy source for growth and development, and are preferentially metabolized for energy production. Nonetheless, excessive intake of SFAs has been linked to elevated cholesterol levels, thereby heightening the risk of atherosclerosis and cardiovascular diseases [25,26]. The study revealed that the SFA profile was predominantly characterized by C16:0 and C18:0, reflecting the typical fatty acid composition observed in the muscle tissue of many crustaceans [19,27]. In the study, the SFA content in NX was the highest, while JL and HH exhibited significantly lower levels of SFA, exhibiting reductions in both C16:0 and C18:0 when compared to other areas. The results indicated that the SFAs content varied with the culture environment, which in turn influenced muscle quality. Previous studies have also shown disparities in the fatty acid composition of *P. clarkii* muscle across various regions, with Hubei exhibiting lower levels of C15:0, C17:0, and C22:0 compared to Hunan and Guangdong [28]. Moreover, there are noticeable differences in the contents of C14:0, C17:0, C20:0, and C24:0 among Suqian, Nanjing, and Suzhou [22].

In this study, PUFAs, predominantly consisted of linoleic acid (C18:2n6) and EPA (C20:5n3), were found in higher content levels as compared to SFAs and MUFAs, indicating that *P. clarkii* from rice fields possess a superior fatty acid nutritional profile. These results aligned with those from previous research on the nutritional composition of *P. clarkii* muscle tissue [29]. PUFA includes n-3 and n-6 PUFA. n-3 PUFAs encompass compounds such as C18:3n3 (α-linolenic acid, ALA), C20:3n3, C20:5n3 (eicosapentaenoic acid, EPA), and C22:6n3 (docosahexaenoic acid, DHA). The n-6 PUFA, including C18:2n6 (linoleic acid, LA), C20:3n6, and C20:4n6 (arachidonic acid, AA), are in metabolic competition with n-3 PUFAs [27]. An insufficient intake of n-3 PUFAs leads to an increase in n-6 PUFA derivatives. LA and AA are critical essential fatty acids (EFAs) indispensable for human health; they play a fundamental role in cellular function and confer benefits such as cardiovascular protection, neuroprotection, anti-osteoporotic, and anti-inflammatory effects [20,30,31]. In this study, the n-3/n-6 PUFA ratio exceeded 1.00 in all regions except for NX (0.92), which suggests that the *P. clarkii* cultivated in the integrated rice–crayfish farming system possess great nutritional quality, thereby rendering them a high-quality source of fatty acids. We also observed that the content of PUFA, n-3 PUFA, and EPA + DHA in FX and SQ were significantly higher, while those in HH and JL were significantly lower, indicating that different cultivation environments have a significant impact on the fatty acid composition of *P. clarkii*, which was similar to the results reported by Jia and Xu et al. [22,28].

### 4.3. The Effects of Farming Condition on Minerals

Minerals are crucial for aquatic animals, providing essential elements and participating in various physiological functions that enhance immunity, promote bone growth, and synthesize hormones and proteins [7,32]. The content of mineral elements is influenced by various factors, such as feed and farming environment. In this experiment, regarding macroelements, the order of concentration was K > Na > Mg > Ca, which was similar to the results of previous studies on crustaceans [33,34]. The concentrations of K and Mg exhibited slight variations across different regions, yet both were predominantly higher in the FX area. Ca plays an important role in physiological processes such as bone and tooth mineralization, hormone regulation, neurotransmitter release, and signal conduction [35]. The content was significantly higher in SQ and NX, but has no difference in other areas.

In this experiment, Zn exhibited the highest trace element content, followed by Fe, Cu, and Mn, respectively, which was similar to the results of previous studies on *P. clarkii* [36]. The abundance of trace elements in organisms is generally positively correlated with the trace elements in environment. Zn and Fe are pivotal elements associated with the physiological or enzymatic activities of aquatic animals. Zn is crucial for nervous system development, enhancing immunity, and carbohydrate and protein metabolism [37]. Fe plays an important role as a coenzyme in the composition of many enzymes such as catalase [38], increases feed utilization, and the nonspecific immunity of crustaceans [39]. Cu is an essential element for the formation of hemocyanin in crustaceans and plays an important role in development and nucleic acid synthesis. The Cu in the environment basically meets the growth demand [7,40]. Excess Cu may cause heavy metal poisoning, reduce antioxidant capacity, and harm health [41]. Mn can enhance immunity by increasing the enzyme activity in the body’s nonspecific immunity, and improve the antioxidant capacity [42]. In the study, the Zn content in NX and HH, Fe content in NX and HH, Cu content in XY, HH, and JL, and Mn content in JL, HH, and QJ were significantly higher than other areas, which indicated the trace elements was significantly influenced by farming environment. The difference in trace elements may affect the nutrient value of muscle in *P. clarkii*.

### 4.4. The Effects of Farming Condition on Antioxidant Capacity

In organisms, the levels of ROS are typically maintained in a dynamic equilibrium, with excessive ROS being neutralized by antioxidant enzyme systems, such as GSH-Px, SOD, and CAT [15]. These enzymes work synergistically to scavenge ROS, with SOD and CAT demonstrating significant antioxidant and ROS scavenging capabilities, thereby mitigating oxidative damage [43,44]. GSH-Px facilitates the decomposition of hydrogen peroxide (H_2_O_2_) via the catalytic oxidative coupling of GSH, a potent antioxidant that can eliminate peroxides and repair damaged cells through reduction processes [45]. In addition, T-AOC can serve as an indicator of the body’s ROS scavenging ability and overall antioxidant potential. Excessive ROS levels can attack unsaturated fatty acids in cell membranes, causing lipid peroxidation [43], which ultimately leads to the formation of MDA [45]. Antioxidant capacity could be affected by many factors. For example, the MDA content of *P. clarkii* in the rice–crayfish co-culture was significantly lower than that in the wild-caught culture, but the SOD and CAT contents had no significant difference [13]. MDA levels were found to increase in aquatic environments with elevated ammonia concentrations, while the activities of antioxidant enzymes, such as SOD, CAT, and GSH-Px, experienced a decrease [46]. Sediment matrices from diverse sources could also significantly affect the antioxidant capacity of *P. clarkii* [47]. This study revealed elevated levels of T-AOC, GSH-Px, and CAT in the YZ, XY, and YY groups. Similarly, increased levels of SOD and GSH were observed in the SQ and XY groups. The enhanced antioxidant capacity is likely to contribute to the crayfish’s resilience against adverse external stimuli. Furthermore, the improved antioxidant capacity in muscle tissue can enhance meat quality by diminishing oxidative stress, which is known to degrade meat quality [47]. However, antioxidant capacity is a complex physiological trait that cannot be fully evaluated using a single biomarker. Therefore, the combined interpretation of antioxidant defense markers and oxidative damage indicators is necessary [14]. In the present study, FX showed relatively high SOD activity, but it also exhibited the highest MDA content, indicating that enhanced SOD activity did not necessarily correspond to reduced lipid peroxidation. Similarly, YY showed high GSH-Px activity but relatively low SOD activity, suggesting differential regulation of antioxidant enzymes. These mixed patterns indicate that the antioxidant status of *P. clarkii* muscle varies among regions.

### 4.5. Regional Variation in the Metabolomic Profiles

The clear clustering of samples according to geographic origin, together with the large number of differentially accumulated metabolites identified, indicates that the metabolome of rice-field crayfish carries a strong regional signature. Previous studies have shown that culture environment, farming mode, and geographic origin can substantially influence the biochemical composition and quality traits of aquatic products, including crayfish [12]. In the present study, FX was selected as the high-quality regional reference because it exhibited superior nutrition, including higher levels of nutritionally valuable amino acids and fatty acids. Previous studies indicated that amino acid composition, fatty acid profiles, mineral elements, and flavor-related compounds are key indicators for evaluating the nutritional and sensory quality of crayfish muscle [12,48]. The broad range of increased and decreased metabolites observed further suggests that regional variation was not confined to a few isolated compounds, but reflected coordinated changes across multiple metabolic modules, particularly amino acid, lipid, and energy metabolism [49].

Amino acid metabolism represented one of the major pathways contributing to regional differentiation. The differential accumulation of amino acid-derived carboxylic acids, aromatic amino acid derivatives, and indole-related metabolites suggests that protein turnover, nitrogen metabolism, and amino acid catabolism differed among regions. These metabolic shifts are relevant to muscle quality because free amino acids and their derivatives contribute directly or indirectly to nutritional value, taste formation, and physiological regulation [12,48]. For example, changes in amino acid-derived compounds may influence umami-related attributes, nitrogen balance, and the availability of bioactive metabolites [50]. The region-specific variation in indole-related metabolites may also reflect differences in tryptophan metabolism and microbial metabolic inputs, because indole derivatives are closely linked to host–microbiota interactions, immune regulation, and redox homeostasis [51,52]. Although individual metabolites varied among regions, the overall pattern indicates that amino acid metabolism may be an important biochemical basis for regional differences in the nutritional and sensory properties of rice-field crayfish. Lipid metabolism showed even more pronounced regional heterogeneity, involving glycerolipids, fatty acyls, steroids, and glycerophospholipids. These lipid classes are closely associated with energy storage, membrane architecture, nutritional value, and stress adaptation [53]. The enrichment of specific glycerolipids in HH, NX, CS, JL, and SQ suggests that lipid deposition and mobilization differed among regional populations. Such variation may be partly related to differences in food resources, rice-field productivity, water temperature, or culture management. Fatty acyls were mainly elevated in YY and NX, indicating that these regions may promote greater accumulation or remodeling of fatty acid-related metabolites. Because fatty acids are important determinants of the nutritional quality and commercial value of aquatic products, these changes may have direct implications for regional quality evaluation and product differentiation [12,48]. In addition, PC and PE are major structural components of biological membranes and are involved in membrane fluidity, lipid transport, and cellular stress responses [54]. Their enrichment in YY, HH, and JL may indicate enhanced membrane biosynthesis or adaptive remodeling of muscle cells under specific environmental conditions. In contrast, the lower abundance of several glycerophospholipids in CS, QJ, and SQ suggests that membrane lipid composition may be regionally constrained or redirected towards other metabolic demands. In ectothermic animals, membrane lipid remodeling is considered an important mechanism for maintaining membrane function and mitochondrial activity under changing environmental conditions [54,55]. The metabolomic findings provided complementary biochemical evidence for the regional differences observed in conventional amino acid and fatty acid analyses. Regions with differences in TAA, EAA, total fatty acids, PUFA, n-3 PUFA, and DHA/EPA also showed region-specific changes in amino acid- and lipid-related metabolites, including carboxylic acids, indole derivatives, glycerolipids, fatty acyls, phosphatidylcholines, and phosphatidylethanolamines. These results suggest that the observed variation in muscle nutritional composition was accompanied by changes in amino acid and lipid metabolic processes, further supporting the regional heterogeneity of *P. clarkii* muscle quality. These results imply that lipid metabolic differences among rice-field crayfish are not limited to nutritional lipid accumulation, but also involve deeper changes in cellular structure and physiological homeostasis. From an applied perspective, these findings highlight the potential of metabolomics as a powerful tool for evaluating regional quality differences in rice-field crayfish and provides a biochemical basis for product grading and geographic origin discrimination.

Several limitations of this study should be acknowledged. First, all samples were collected from integrated rice–crayfish farming systems, without parallel samples from commercial pond-based farms; therefore, this study cannot directly compare growth performance, survival rate, stocking density, muscle quality, or antioxidant capacity between rice paddy systems and other culture models. Second, environmental parameters, including water quality, temperature, soil properties, and background mineral levels, were not measured. Thus, although clear regional differences were observed in the nutritional composition, antioxidant-related biomarkers, and metabolomic profiles of *P. clarkii* muscle, these differences cannot be attributed to specific environmental drivers. Future studies should combine muscle quality assessment with commercial farm controls, systematic environmental monitoring, and farming-management records to clarify the mechanisms underlying regional variation in crayfish quality.

## 5. Conclusions

This study assessed the nutritional profiles, antioxidant capacity, and metabolomic profiles of *P. clarkii* across different regions, uncovering significant regional variations in amino acid, PUFA, and antioxidant capacity. Notably, specimens from FX and SQ regions displayed elevated levels of amino acids and PUFAs. Furthermore, trace elements were found to be more abundant in specimens from JL and HH. In terms of antioxidant activity, XY and YZ specimens showed higher enzyme activities. Together, these findings reveal substantial regional heterogeneity in the nutritional quality, metabolic features, and antioxidant capacity of *P. clarkii*. Our study provides a scientific basis for the quality evaluation of rice-field cultured crayfish and offers practical guidance for developing region-specific, high-value crayfish products.

## Figures and Tables

**Table 1:** Amino acid composition of *P. clarkii* muscles from different areas (dry matter; *n* = 10; g/100 g).

Amino Acids	FX	SQ	YZ	XY	QJ	JL	HH	YY	^C^S	NX
Met ^#^	1.62 ± 0.12 ^a^	1.42 ± 0.06 ^abc^	1.29 ± 0.03 ^bcd^	1.35 ± 0.06 ^bcd^	1.26 ± 0.03 ^cd^	1.17 ± 0.08 ^d^	1.24 ± 0.08 ^cd^	1.29 ± 0.09 ^bcd^	1.49 ± 0.06 ^ab^	1.57 ± 0.03 ^a^
Thr ^#^	2.72 ± 0.14 ^a^	2.46 ± 0.10 ^ab^	2.40 ± 0.03 ^b^	2.47 ± 0.12 ^ab^	2.42 ± 0.04 ^ab^	2.30 ± 0.13 ^b^	2.23 ± 0.06 ^b^	2.34 ± 0.14 ^b^	2.60 ± 0.17 ^ab^	2.55 ± 0.11 ^ab^
Phe ^#^	2.89 ± 0.16 ^a^	2.68 ± 0.11 ^a^	2.54 ± 0.03 ^b^	2.68 ± 0.13 ^a^	2.60 ± 0.05 ^a^	2.56 ± 0.16 ^a^	2.47 ± 0.08 ^b^	2.56 ± 0.15 ^a^	2.77 ± 0.17 ^a^	2.65 ± 0.06 ^a^
IIe ^#^	3.10 ± 0.17 ^a^	2.86 ± 0.12 ^a^	2.69 ± 0.04 ^b^	2.89 ± 0.14 ^a^	2.75 ± 0.06 ^a^	2.75 ± 0.19 ^a^	2.65 ± 0.07 ^b^	2.76 ± 0.17 ^a^	2.89 ± 0.19 ^a^	2.80 ± 0.05 ^a^
Val ^#^	3.16 ± 0.17 ^a^	2.95 ± 0.12 ^a^	2.76 ± 0.04 ^b^	2.94 ± 0.14 ^a^	2.80 ± 0.06 ^a^	2.80 ± 0.19 ^a^	2.71 ± 0.08 ^b^	2.82 ± 0.17 ^a^	2.96 ± 0.19 ^a^	2.91 ± 0.06 ^a^
Leu ^#^	5.74 ± 0.32 ^a^	5.17 ± 0.20 ^ab^	4.93 ± 0.06 ^b^	5.19 ± 0.25 ^ab^	5.01 ± 0.10 ^b^	4.88 ± 0.30 ^b^	4.75 ± 0.13 ^b^	4.96 ± 0.30 ^b^	5.38 ± 0.33 ^ab^	5.19 ± 0.10 ^ab^
Lys ^#^	5.94 ± 0.32 ^a^	5.45 ± 0.25 ^ab^	5.10 ± 0.06 ^bc^	5.29 ± 0.27 ^abc^	5.23 ± 0.09 ^bc^	5.00 ± 0.31 ^bc^	4.70 ± 0.15 ^c^	5.10 ± 0.29 ^bc^	5.45 ± 0.37 ^ab^	5.32 ± 0.13 ^abc^
His	1.61 ± 0.08 ^a^	1.45 ± 0.07 ^ab^	1.27 ± 0.02 ^cd^	1.33 ± 0.06 ^bcd^	1.26 ± 0.02 ^cd^	1.28 ± 0.08 ^cd^	1.17 ± 0.03 ^d^	1.30 ± 0.08 ^bcd^	1.38 ± 0.09 ^bc^	1.33 ± 0.03 ^bcd^
Arg	8.09 ± 0.36 ^a^	7.70 ± 0.34 ^ab^	7.43 ± 0.08 ^ab^	7.50 ± 0.34 ^ab^	7.56 ± 0.14 ^ab^	7.06 ± 0.39 ^b^	7.01 ± 0.17 ^b^	6.99 ± 0.38 ^b^	7.84 ± 0.53 ^ab^	7.80 ± 0.20 ^ab^
Cys	0.80 ± 0.10 ^ab^	0.81 ± 0.09 ^a^	0.56 ± 0.01 ^cde^	0.61 ± 0.07 ^bcd^	0.55 ± 0.08 ^de^	0.51 ± 0.06 ^de^	0.41 ± 0.04 ^e^	0.53 ± 0.06 ^de^	0.65 ± 0.06 ^abcd^	0.75 ± 0.04 ^abc^
Pro	2.35 ± 0.14 ^a^	2.13 ± 0.09 ^abc^	2.02 ± 0.04 ^bcd^	2.15 ± 0.09 ^ab^	1.99 ± 0.04 ^bcd^	1.87 ± 0.10 ^cd^	1.76 ± 0.06 ^d^	1.89 ± 0.12 ^bcd^	2.14 ± 0.14 ^abc^	2.10 ± 0.06 ^abc^
Ser	2.40 ± 0.11 ^a^	2.09 ± 0.07 ^bcd^	2.06 ± 0.03 ^bcd^	2.08 ± 0.10 ^bcd^	2.09 ± 0.02 ^bcd^	1.97 ± 0.11 ^cd^	1.90 ± 0.06 ^d^	1.99 ± 0.11 ^cd^	2.27 ± 0.14 ^ab^	2.20 ± 0.05 ^abc^
Tyr	2.49 ± 0.16 ^a^	2.18 ± 0.09 ^bcd^	1.97 ± 0.02 ^de^	2.08 ± 0.10 ^cde^	2.09 ± 0.04 ^cde^	1.96 ± 0.11 ^de^	1.90 ± 0.07 ^e^	2.00 ± 0.13 ^de^	2.31 ± 0.12 ^abc^	2.45 ± 0.05 ^ab^
Gly	3.62 ± 0.15 ^a^	2.96 ± 0.11 ^b^	2.51 ± 0.05 ^de^	2.58 ± 0.11 ^cde^	2.91 ± 0.09 ^bc^	2.63 ± 0.16 ^bcde^	2.33 ± 0.07 ^e^	2.65 ± 0.14 ^bcde^	2.77 ± 0.34 ^bcd^	2.67 ± 0.15 ^bcde^
Ala	4.43 ± 0.25 ^a^	3.81 ± 0.14 ^b^	3.78 ± 0.07 ^b^	3.98 ± 0.15 ^ab^	3.74 ± 0.08 ^b^	3.62 ± 0.23 ^b^	3.61 ± 0.08 ^b^	3.62 ± 0.22 ^b^	4.01 ± 0.25 ^ab^	3.98 ± 0.15 ^ab^
Asp	7.12 ± 0.37 ^a^	6.51 ± 0.30 ^ab^	6.03 ± 0.08 ^bc^	6.26 ± 0.34 ^bc^	6.14 ± 0.12 ^bc^	5.96 ± 0.36 ^bc^	5.66 ± 0.19 ^bc^	6.03 ± 0.34 ^bc^	6.60 ± 0.42 ^ab^	6.24 ± 0.15 ^bc^
Glu	10.51 ± 0.61 ^a^	9.34 ± 0.40 ^ab^	8.83 ± 0.13 ^b^	9.32 ± 0.58 ^ab^	8.75 ± 0.17 ^b^	8.56 ± 0.48 ^b^	7.96 ± 0.35 ^bc^	8.71 ± 0.46 ^b^	9.69 ± 0.57 ^ab^	8.94 ± 0.15 ^b^
∑TAA	68.59 ± 3.72 ^a^	61.95 ± 2.64 ^abc^	58.15 ± 0.80 ^bc^	60.68 ± 3.03 ^bc^	59.14 ± 1.07 ^bc^	56.85 ± 3.38 ^bc^	54.43 ± 1.70 ^c^	57.51 ± 3.29 ^bc^	63.18 ± 3.93 ^ab^	61.41 ± 1.33 ^abc^
∑EAA	25.17 ± 1.40 ^a^	22.97 ± 0.95 ^ab^	21.70 ± 0.29 ^b^	22.79 ± 1.10 ^ab^	22.06 ± 0.43 ^b^	21.45 ± 1.35 ^b^	20.74 ± 0.62 ^b^	21.82 ± 1.30 ^b^	23.52 ± 1.47 ^ab^	22.97 ± 0.47 ^ab^
∑EAA/∑TAA	36.70%	37.09%	37.31%	37.56%	37.30%	37.71%	38.10%	37.92%	37.23%	37.41%

Note: ^#^ represents essential amino acids; ΣTAA means total content of amino acids (TAA); ΣEAA means total content of essential amino acids (EAA); ∑EAA/∑TAA means the percentage of EAA in TAA. Values (mean ± SE) in the same row with different letters indicated significant difference (*p* < 0.05). FX, Feixi County; SQ, Suqian city; YZ, Yangzhou city; XY, Xuyi County; QJ, Qianjiang city; JL, Jianli County; HH, Honghu city; YY, Yueyang city; CS, Changsha city; NX, Nanxian County. One-way ANOVA is used for analysis.

**Table 2:** Fatty acid composition of *P. clarkii* muscles from different areas (dry matter; *n* = 10; mg/10 g).

Fatty Acids	FX	SQ	YZ	XY	QJ	JL	HH	YY	CS	NX
C14:0	0.78 ± 0.04 ^a^	0.61 ± 0.02 ^ab^	0.51 ± 0.06 ^bc^	0.49 ± 0.02 ^bc^	0.53 ± 0.05 ^b^	0.31 ± 0.11 ^cd^	0.22 ± 0.13 ^d^	0.48 ± 0.05 ^bc^	0.45 ± 0.03 ^bc^	0.51 ± 0.03 ^bc^
C15:0	1.33 ± 0.06 ^b^	0.89 ± 0.01 ^ef^	1.20 ± 0.05 ^bcd^	1.31 ± 0.01 ^bc^	1.20 ± 0.06 ^bcd^	0.75 ± 0.03 ^f^	1.04 ± 0.13 ^de^	1.15 ± 0.03 ^cd^	0.91 ± 0.01 ^ef^	1.52 ± 0.03 ^a^
C16:0	33.22 ± 0.73 ^a^	31.44 ± 0.61 ^ab^	28.42 ± 1.22 ^b^	31.39 ± 0.35 ^ab^	30.06 ± 0.96 ^ab^	21.99 ± 1.03 ^c^	23.54 ± 3.31 ^c^	32.20 ± 0.82 ^ab^	28.35 ± 0.21 ^b^	30.79 ± 0.31 ^ab^
C17:0	2.10 ± 0.03 ^abc^	1.79 ± 0.03 ^d^	2.18 ± 0.08 ^ab^	2.01 ± 0.03 ^bcd^	2.13 ± 0.06 ^abc^	1.29 ± 0.03 ^e^	1.87 ± 0.25 ^cd^	1.96 ± 0.05 ^bcd^	1.86 ± 0.04 ^cd^	2.37 ± 0.01 ^a^
C18:0	19.54 ± 0.34 ^bc^	20.43 ± 0.48 ^bc^	19.34 ± 0.89 ^bc^	22.60 ± 0.46 ^ab^	20.78 ± 0.88 ^abc^	13.60 ± 0.36 ^d^	18.86 ± 2.94 ^c^	21.62 ± 0.75 ^abc^	21.82 ± 0.37 ^abc^	24.14 ± 0.43 ^a^
C20:0	2.83 ± 0.05 ^cd^	2.76 ± 0.07 ^d^	2.78 ± 0.13 ^cd^	3.41 ± 0.07 ^ab^	3.12 ± 0.10 ^bcd^	2.28 ± 0.09 ^e^	3.08 ± 0.38 ^bcd^	3.14 ± 0.12 ^bcd^	3.27 ± 0.04 ^abc^	3.68 ± 0.14 ^a^
C21:0	0.50 ± 0.02 ^c^	0.52 ± 0.04 ^c^	0.76 ± 0.03 ^b^	0.73 ± 0.03 ^b^	0.65 ± 0.06 ^bc^	0.27 ± 0.09 ^d^	0.96 ± 0.14 ^a^	0.56 ± 0.03 ^bc^	0.56 ± 0.02 ^bc^	0.75 ± 0.04 ^b^
C22:0	2.54 ± 0.02 ^bc^	2.41 ± 0.07 ^bc^	2.28 ± 0.15 ^cd^	3.01 ± 0.10 ^a^	2.49 ± 0.12 ^bc^	1.98 ± 0.06 ^d^	2.52 ± 0.28 ^bc^	2.42 ± 0.11 ^bc^	2.62 ± 0.07 ^bc^	2.81 ± 0.07 ^ab^
C23:0	0.69 ± 0.07 ^bc^	0.56 ± 0.02 ^b^	0.82 ± 0.05 ^ab^	0.77 ± 0.04 ^ab^	0.83 ± 0.05 ^ab^	0.56 ± 0.03 ^c^	0.84 ± 0.09 ^ab^	0.76 ± 0.04 ^ab^	0.75 ± 0.02 ^ab^	0.90 ± 0.01 ^a^
C16:1	8.09 ± 0.26 ^a^	6.06 ± 0.32 ^b^	4.80 ± 0.27 ^cd^	6.34 ± 0.10 ^b^	4.10 ± 0.13 ^d^	4.16 ± 0.33 ^d^	3.17 ± 0.53 ^e^	4.86 ± 0.11 ^cd^	5.20 ± 0.14 ^c^	3.24 ± 0.06 ^e^
C18:1n9c	43.01 ± 0.80 ^ab^	45.26 ± 1.03 ^a^	32.33 ± 1.40 ^c^	38.57 ± 0.45 ^b^	44.02 ± 1.62 ^a^	30.97 ± 1.16 ^c^	33.02 ± 4.28 ^c^	40.84 ± 1.22 ^ab^	42.49 ± 0.57 ^ab^	45.10 ± 0.74 ^a^
C20:1	2.20 ± 0.05 ^de^	1.70 ± 0.05 ^f^	1.84 ± 0.07 ^f^	2.01 ± 0.05 ^ef^	2.67 ± 0.11 ^bc^	1.31 ± 0.05^g^	2.19 ± 0.27 ^de^	2.40 ± 0.12 ^cd^	3.06 ± 0.05 ^a^	2.88 ± 0.10 ^ab^
C22:1n9	1.93 ± 0.07 ^ab^	1.43 ± 0.17 ^c^	1.61 ± 0.18 ^bc^	1.62 ± 0.07 ^bc^	2.30 ± 0.18 ^a^	1.94 ± 0.10 ^ab^	2.07 ± 0.16 ^a^	1.89 ± 0.21 ^ab^	2.19 ± 0.18 ^a^	1.57 ± 0.13 ^bc^
C20:2	2.20 ± 0.03 ^c^	2.25 ± 0.05 ^c^	2.83 ± 0.11 ^b^	2.96 ± 0.06 ^b^	2.69 ± 0.07 ^b^	2.02 ± 0.05 ^c^	2.15 ± 0.33 ^c^	2.71 ± 0.08 ^b^	2.34 ± 0.04 ^c^	3.42 ± 0.04 ^a^
C18:2n6	19.80 ± 0.99 ^a^	20.69 ± 0.48 ^a^	16.68 ± 0.63 ^bc^	20.28 ± 0.19 ^a^	19.07 ± 0.60 ^ab^	13.10 ± 0.66 ^d^	13.82 ± 2.25 ^d^	19.93 ± 0.43 ^a^	15.68 ± 0.26 ^cd^	20.05 ± 0.13 ^a^
C20:3n6	0.57 ± 0.06 ^abc^	0.47 ± 0.02 ^c^	0.69 ± 0.04 ^a^	0.47 ± 0.03 ^c^	0.51 ± 0.04 ^bc^	0.45 ± 0.05 ^c^	0.09 ± 0.09 ^d^	0.56 ± 0.05 ^abc^	0.48 ± 0.01 ^c^	0.63 ± 0.04 ^ab^
C20:4n6	9.73 ± 0.11 ^cde^	9.44 ± 0.14 ^de^	12.21 ± 0.43 ^b^	8.01 ± 0.11 ^f^	10.88 ± 0.31 ^c^	7.30 ± 0.07 ^f^	8.61 ± 1.10 ^ef^	10.28 ± 0.29 ^cd^	8.45 ± 0.14 ^ef^	13.60 ± 0.41 ^a^
C18:3n3	8.96 ± 0.33 ^a^	6.03 ± 0.11 ^b^	6.61 ± 0.28 ^b^	6.33 ± 0.15 ^b^	4.81 ± 0.14 ^cd^	3.63 ± 0.12 ^e^	4.73 ± 0.99 ^cde^	4.57 ± 0.05 ^de^	5.70 ± 0.09 ^bc^	4.47 ± 0.15 ^de^
C20:3n3	1.41 ± 0.04 ^b^	1.13 ± 0.04 ^bc^	1.32 ± 0.06 ^b^	1.65 ± 0.05 ^a^	1.00 ± 0.05 ^cd^	0.75 ^d^	1.13 ± 0.25 ^bc^	0.92 ± 0.02 ^cd^	1.35 ± 0.03 ^b^	1.33 ± 0.05 ^b^
C20:5n3	24.61 ± 0.39 ^a^	23.88 ± 0.38 ^a^	15.96 ± 0.68 ^e^	21.49 ± 0.30 ^bc^	18.93 ± 0.59 ^d^	13.14 ± 0.12 ^f^	13.22 ± 1.53 ^f^	22.72 ± 0.85 ^ab^	19.89 ± 0.31 ^cd^	18.31 ± 0.24 ^d^
C22:6n3	5.45 ± 0.08 ^e^	8.42 ± 0.22 ^a^	6.12 ± 0.26 ^de^	6.74 ± 0.10 ^d^	6.84 ± 0.22 ^cd^	3.80 ± 0.06 ^f^	4.46 ± 0.52 ^f^	7.70 ± 0.23 ^b^	8.01 ± 0.21 ^ab^	7.51 ± 0.13 ^bc^
∑FA	191.46 ± 3.19 ^a^	188.14 ± 3.96 ^a^	161.25 ± 6.76 ^bc^	182.16 ± 2.16 ^ab^	179.59 ± 6.00 ^ab^	125.56 ± 4.17 ^d^	141.57 ± 19.55 ^cd^	183.62 ± 5.03 ^ab^	175.39 ± 2.13 ^ab^	189.53 ± 1.69 ^a^
∑SFA	63.52 ± 1.17 ^a^	61.39 ± 1.27 ^ab^	58.28 ± 2.60 ^ab^	65.72 ± 0.95 ^a^	61.79 ± 2.17 ^ab^	43.03 ± 1.63 ^c^	52.92 ± 7.63 ^b^	64.27 ± 1.97 ^a^	60.56 ± 0.71 ^ab^	67.44 ± 0.99 ^a^
∑MUFA	55.22 ± 1.12 ^a^	54.44 ± 1.47 ^a^	40.58 ± 1.83 ^b^	48.53 ± 0.49 ^a^	53.08 ± 1.98 ^a^	38.37 ± 1.52 ^b^	40.44 ± 5.14 ^b^	49.98 ± 1.41 ^a^	52.94 ± 0.84 ^a^	52.78 ± 0.79 ^a^
∑PUFA	72.71 ± 1.04 ^a^	72.30 ± 1.34 ^a^	62.40 ± 2.40 ^b^	67.92 ± 0.82 ^ab^	64.73 ± 1.95 ^ab^	44.17 ± 1.07 ^c^	48.21 ± 6.93 ^c^	69.37 ± 1.71 ^ab^	61.89 ± 0.70 ^b^	69.31 ± 0.76 ^ab^
∑n-3PUFA	40.42 ± 0.24 ^a^	39.46 ± 0.69 ^ab^	30.00 ± 1.22 ^e^	36.21 ± 0.57 ^bc^	31.58 ± 0.99 ^de^	21.31 ± 0.29 ^f^	23.54 ± 3.21 ^f^	35.90 ± 1.05 ^bc^	34.95 ± 0.40 ^cd^	31.61 ± 0.50 ^de^
∑n-6PUFA	30.09 ± 1.05 ^b^	30.59 ± 0.61 ^ab^	29.57 ± 1.08 ^b^	28.76 ± 0.25 ^b^	30.46 ± 0.91 ^ab^	20.84 ± 0.75 ^c^	22.52 ± 3.39 ^c^	30.77 ± 0.67 ^ab^	24.61 ± 0.38 ^c^	34.28 ± 0.35 ^a^
n-3/n-6	1.35	1.29	1.01	1.26	1.04	1.03	1.05	1.17	1.42	0.92
DHA + EPA	30.06 ± 0.42 ^ab^	32.30 ± 0.57 ^a^	22.08 ± 0.92 ^d^	28.23 ± 0.40 ^bc^	25.77 ± 0.81 ^c^	16.94 ± 0.17 ^e^	17.68 ± 2.04 ^e^	30.42 ± 1.06 ^ab^	27.90 ± 0.42 ^bc^	25.82 ± 0.33 ^c^

Note: ∑FA means total content of fatty acids, ∑SFA means total content of saturated fatty acids, ∑MUFA means total content of monounsaturated fatty acid, ∑PUFA means total content of polyunsaturated fatty acids; DHA + EPA means the content of C20:5n3 and C22:6n3; values (mean ± SE) in the same row with different letters indicated significant difference (*p* < 0.05). FX, Feixi County; SQ, Suqian city; YZ, Yangzhou city; XY, Xuyi County; QJ, Qianjiang city; JL, Jianli County; HH, Honghu city; YY, Yueyang city; CS, Changsha city; NX, Nanxian County. One-way ANOVA is used for analysis.

## Data Availability

Data is contained within the article or the Supplementary Materials; original data can be obtained by contacting the authors.

## Supplementary Materials

The following supporting information can be downloaded at: https://www.mdpi.com/article/10.3390/antiox15070887/s1. Figure S1. Assessment of quality of metabolomics data; Methods of LC–MS analysis; Figure S2. KEGG pathway enrichment of differential metabolites; Figure S3. Bubble plot of enriched metabolic pathways in samples from different regions; Figure S4. Regional variation in glycerophospholipid metabolites.
