# Supplementary material for: Comparison of Nutritional Profiles and Antioxidant Capacity in the Muscle of Procambarus clarkii Farmed Across Various Rice Paddy Regions in Eastern China
Source: Antioxidants (Basel). 2026 Jul 17;15(7):887. doi: 10.3390/antiox15070887 (PMC13404522; doi:10.3390/antiox15070887)
Supplement: Supplementary file 1 [file antioxidants-15-00887-s001.zip › antioxidants-4358276-supplementary.pdf]

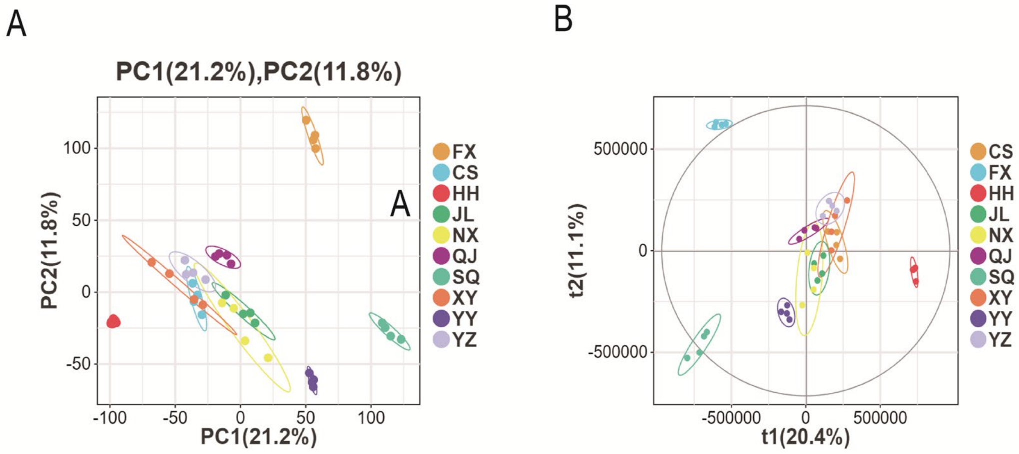

Figure S1. Assessment of quality of metabolomics data. (A) PCA score plot assessing quality of metabolomics data; (B) Partial Least Squares Discriminant Analysis (PLS-DA). The QC samples exhibit tight clustering in both plots, indicating that the detection method is stable and the data is reliable.

LC–MS analysis

Metabolite separation was performed using an Agilent 1290 Infinity LC System ultra-high-performance liquid chromatography (UHPLC) system equipped with an ACQUITY UPLC BEH Amide Column HILIC column. The flow rate was set at 0.5 mL min^{-1}, the column temperature was maintained at 25 °C, and the injection volume was 2 μL. Metabolites were separated using a gradient elution program with mobile phase A consisting of water containing 25 mM ammonium acetate and 25 mM ammonia solution, and mobile phase B consisting of acetonitrile. The gradient program was as follows: 95% B for the initial 0.5 min; a linear decrease from 95% to 40% B from 0.5 to 9 min, followed by maintenance at 40% B for 1 min; B was then increased from 40% to 95% from 9 to 12 min and maintained for an additional 2.9 min for column equilibration. Throughout the analytical process, samples were maintained at 4 °C in an autosampler. Mass spectrometric data were acquired using an TripleTOF 6600 System mass spectrometer (SCIEX). The electrospray ionization voltage was set to 5.5 kV, the ion source gas 1 radio frequency level was set to 60, and the source temperature was maintained at 600°C. Mass spectra were collected over an m/z range of 60-1000.

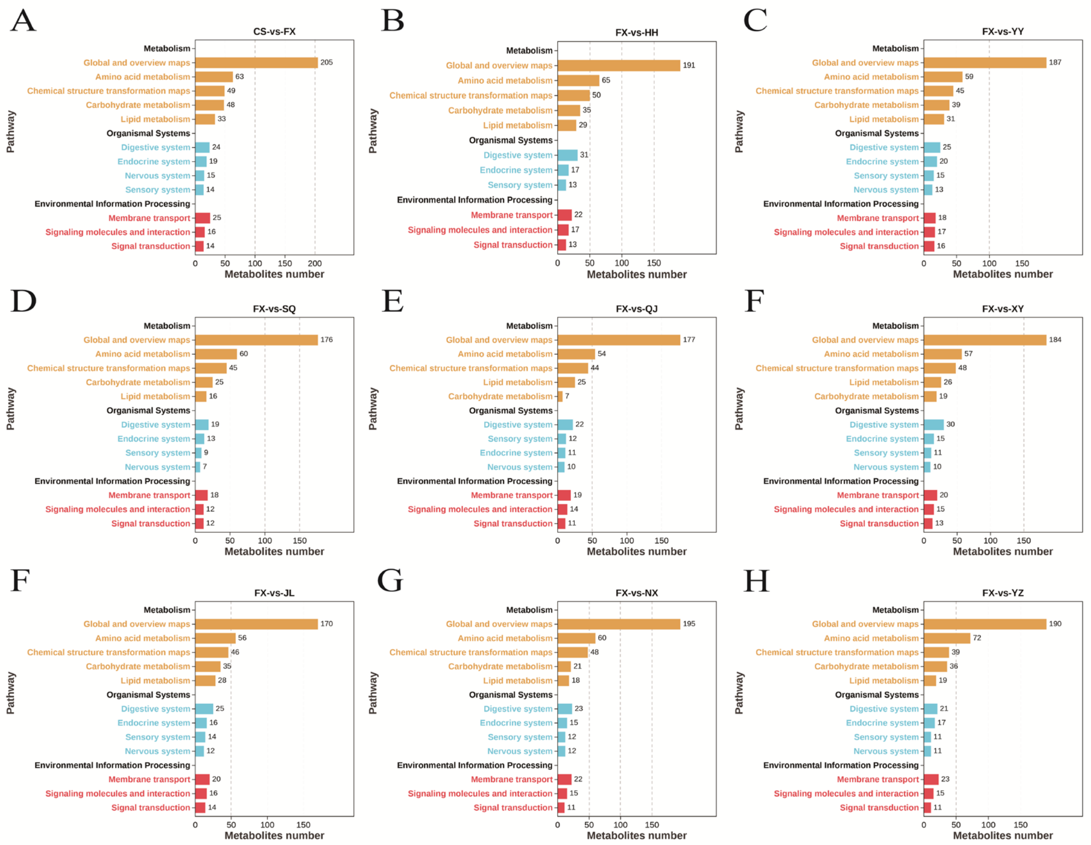

Figure S2. KEGG pathway enrichment of differential metabolites. (A)-(I) show pathway enrichment of differential metabolites identified between FX and each of the other sampling sites. Bubble size represents the number of differential metabolites enriched in each pathway, whereas bubble colour indicates the statistical significance of pathway enrichment, with higher values denoting greater enrichment significance.

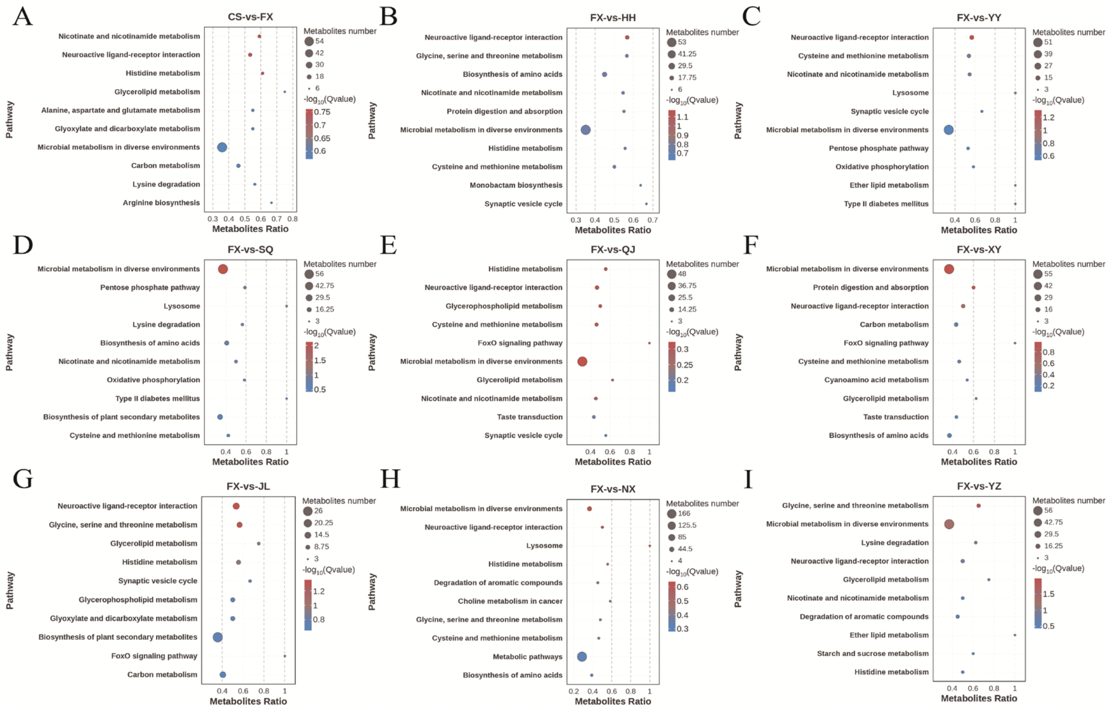

Figure S3. Bubble plot of enriched metabolic pathways in samples from different regions. (A)-(I) display significantly enriched pathways for differential metabolites in pairwise comparisons between FX and the other sampling sites. The x-axis represents the number of metabolites assigned to each pathway, while the y-axis denotes KEGG level A and B pathway categories.

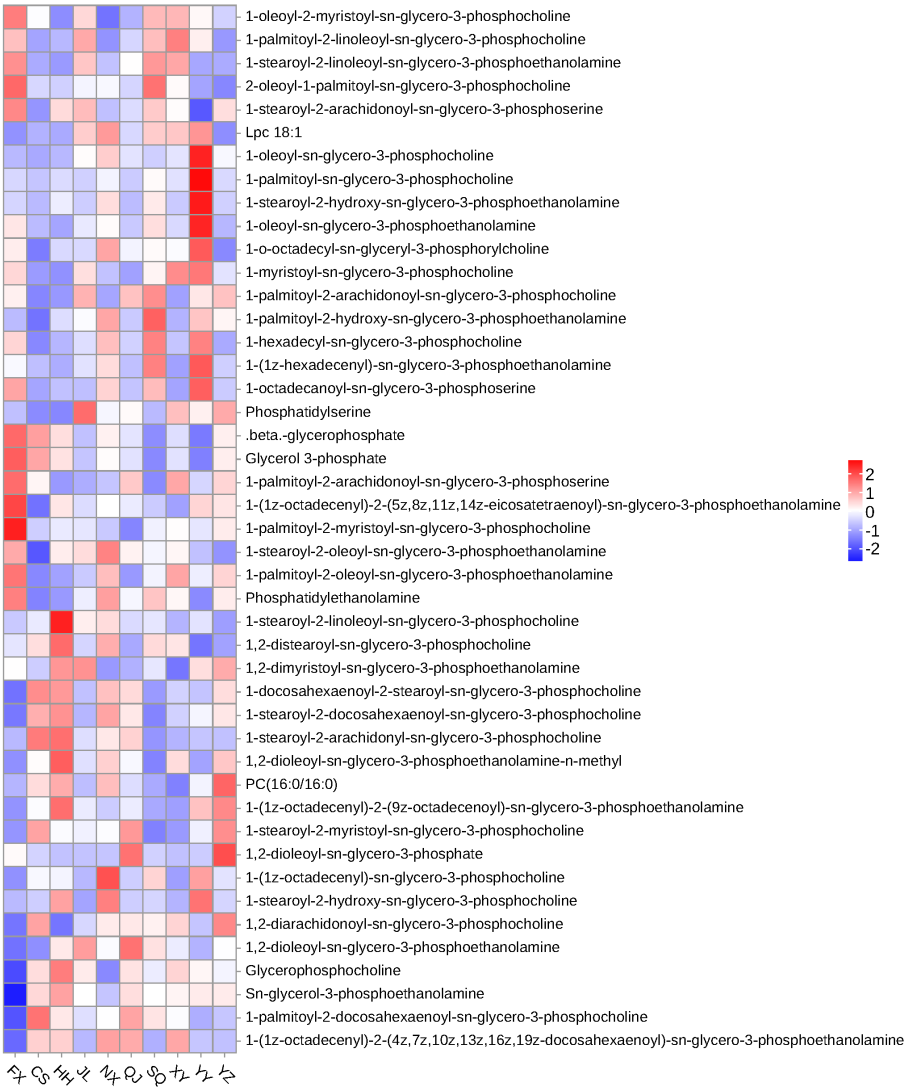

Figure S4. Regional variation in glycerophospholipid metabolites. Heatmap showing the relative abundance patterns of glycerophospholipid-related metabolites across ten sampling regions. Each row represents an annotated glycerophospholipid metabolite while each column represents a regional sample group. Color gradient indicate scaled relative metabolite abundance, with red denoting higher abundance and blue denoting lower abundance.
